# Exploring the Link Between Inflammatory Gene Variants and Phenotypes of Periodontal Disease and Obstructive Sleep Apnea

**DOI:** 10.1055/s-0046-1829075

**Published:** 2026-10-09

**Authors:** Lauren Sofia Yepes Fernández, Mariana Almonacid Garzón, Mayra Alexandra Téllez Corral, Francisco Sir-Mendoza, Liliana Otero, Mauricio Rey Buitrago

**Affiliations:** 1 Department of Biology, Sciences Faculty, Universidad Nacional de Colombia, Colombia; 2 Health Sciences Faculty, Universidad Colegio Mayor de Cundinamarca, Colombia; 3 Dental Center Research, Dentistry Faculty, Pontificia Universidad Javeriana, Colombia; 4 Instituto de Genética, Medicine Faculty, Universidad Nacional de Colombia, Colombia

**Keywords:** IL-1B, IL-10, IL-17, inflammatory response, periodontitis, obstructive sleep apnea

## Abstract

Periodontal disease (PD) and obstructive sleep apnea (OSA) are prevalent chronic inflammatory conditions affecting over 50% of Colombians and a substantial proportion of the global population. This study investigated genetic associations between interleukin (IL) gene variants (IL-1β, IL-10, IL-17) and the risk of PD, OSA, or their coexistence in 129 Colombian individuals, including cases (PD, OSA, PD + OSA) and healthy controls. Genotyping was performed using PCR amplification and Sanger sequencing. Key findings revealed that IL-1β variants (rs1143627, rs16944) significantly increased OSA risk (OR = 3.6 and 3.36; *p* = 0.004 and 0.008). The IL-10 (rs1800896) also elevated OSA risk (OR = 4.44; *p* = 0.004), whereas the IL-17 (rs2275913) showed a protective effect (OR = 0.21; *p* = 0.0003). Among individuals with PD + OSA, IL-1β variants (rs1143627, rs16944) conferred an even higher risk (OR = 4.8; *p* = 0.0007–0.0008). No significant genetic associations were found for PD alone. In conclusion, these findings highlight the central role of inflammation in PD-OSA comorbidity, driven by dysregulated interleukin signaling pathways. Variants in IL-1β and IL-10 may predispose individuals to both conditions, suggesting shared inflammatory mechanisms. These insights could inform the development of targeted therapies addressing inflammatory pathways underlying PD and OSA. The study underscores the importance of genetic and molecular research in advancing personalized treatment strategies for overlapping chronic diseases.

## Introduction

Periodontal disease (PD) is a chronic, infectious-inflammatory condition characterized by the progressive destruction of tooth-supporting tissues, leading to the loss of periodontal attachment and alveolar bone. 1 Its causes include bacterial plaque, non-modifiable genetic factors, and modifiable risk factors such as smoking and poor hygiene habits. 1 2 PD is the primary cause of tooth loss and the most common inflammatory disease in adults. The Centers for Disease Control and Prevention (CDC) describe PD as a global pandemic, affecting speech, self-esteem, and quality of life. Its prevalence ranges from 20% to 50% of the global population, with developing and developed countries equally affected. In Colombia, a national health survey reported that 61.8% of the population suffers from PD. 2 3 4 5

The chronic inflammation associated with PD has prompted research into its potential links with other diseases. Over time, connections between PD and conditions such as cancer, type 2 diabetes, and neurodegenerative diseases have been established. 6 7 More recently, studies have begun exploring its relationship with obstructive sleep apnea (OSA), a sleep respiratory disorder characterized by repeated episodes of upper airway collapse during sleep, often leading to oxygen desaturation and microarousals. 8 9

OSA is highly prevalent in adults, particularly among obese individuals. It has been established that OSA increases the risk of cardiovascular disease and metabolic disorders, including diabetes, hypertension, heart disease, and stroke. 10 11 Additionally, it has been shown that individuals with both PD and OSA exhibit worse periodontal health, including deeper probing depths, increased clinical attachment loss, and higher percentages of biofilm. These individuals also have a higher apnea-hypopnea index (AHI), reflecting more than 15 events of apnea or hypopnea per hour, and often experience oral dryness due to mouth breathing. 11 12 13 Moreover, men with severe OSA are more likely to have advanced PD (Stage III). 14 15

The pathophysiological mechanisms involved in the PD and OSA relationship are not yet fully understood, but alterations in the oral microbiota of individuals with PD and OSA and inflammatory events have been proposed. 16 17 18 Genetic factors may play a role, as studies have begun investigating how certain genetic variants could contribute to the co-occurrence of PD and OSA. 15 One gene of interest is interleukin-10 (IL-10), a cytokine produced by lymphocytes and monocytes. IL-10 has both immunomodulatory and anti-inflammatory functions, suppressing cellular immune responses. 14 15 16 17 18 19 It can inhibit the production of tumor necrosis factor (TNF-α) and interleukin-6 (IL-6), which are both involved in inflammation and tissue destruction in chronic periodontitis. 20 IL-10 has been found to protect against bone loss by suppressing these destructive processes. Variants in the promoter region of the IL-10 gene, particularly at positions -1082, -819, and -592, are associated with higher levels of IL-10, which is protective against PD. Although these genetic variants have not been directly linked to OSA, some evidence suggests that IL-10 may influence breathing patterns, and that IL-10 receptors in the brain's microglia could play a role in regulating respiratory rhythms. 21 22 23 24 25 26 27

Another cytokine of interest is interleukin-17 (IL-17), which plays a key role in inflammatory diseases, including PD. The genes encoding IL-17A and IL-17F are located on chromosome 6, and polymorphisms in these genes have been linked to changes in gene expression and susceptibility to various inflammatory conditions, such as psoriasis, ulcerative colitis, and rheumatoid arthritis. Specific variants in the IL-17A gene, like the G/A variant at position -197, and the T/C variant in IL-17F, have been associated with PD. Research into these polymorphisms has also begun exploring their possible connection to OSA, though this area remains less studied. 28 29 30 31 32

IL-1B is a proinflammatory cytokine involved in the regulation of inflammation in adults with OSA. Candidate cytokines genes have been identified as possible susceptibility loci, such as *IL-1β* . Several SNVs located in the promoter regions (c-511C > T; rs16944 and c.-31T > C; rs1143627) have been reported to participate in systemic and local inflammatory processes that may predispose individuals to OSA and PD. However, genotype-phenotype association studies show inconsistent results across populations, likely due to ethnic and regional differences, as well as the multifactorial and polygenic nature of these conditions. Furthermore, studies exploring the correlation between these phenotypes (PD and OSA) remain largely descriptive, and molecular investigations have typically been conducted for each pathology separately. 33 34 35 36

In summary, both PD and OSA are chronic inflammatory conditions with overlapping risk factors and possible shared mechanisms. While genetic research is still in its early stages, emerging studies suggest that certain cytokines and genetic polymorphisms may help explain the relationship between these two conditions.

Considering this background, which suggests that SNVs in these genes may be involved in the development and progression of PD and OSA, it is pertinent to explore this relationship. Therefore, this preliminary study, based on a small but well-characterized sample, was designed to investigate the association of IL-1β (rs16944, rs1143627), IL-10 (rs1800872, rs1800871, rs1800896), and IL-17 (rs2275913, rs763780) with PD and OSA in a sample of the Colombian population.

## Materials and Methods

### Study Design and Participants

A non-probabilistic, observational, case-control, analytical study was conducted. **The sample size was based on convenience sampling.** Case subjects ( *n* = 65) were divided into the following groups: those with a clinical diagnosis of PD without OSA, those with a clinical diagnosis of OSA without PD, and those characterized by having both conditions, OSA and PD. Control subjects ( *n* = 64) were not diagnosed with either disease—neither OSA nor PD—and were matched for age and sex. The target distribution for the study design aimed for ~ 30 subjects in each subgroup, i.e., controls, PD, OSA, and PD + OSA. For the selection of subjects with PD, the diagnosis was made by an expert professor from the postgraduate program in periodontics: a full-mouth periodontal examination was performed based on the criteria of the latest classification system for periodontal diseases and conditions defined by the American Academy of Periodontology (AAP) and the European Federation of Periodontology (EFP). The clinical parameters included periodontal pocket depth (PD > 3 mm on two or more teeth), infrabony and furcation defects, hypermobility, and bleeding.

Regarding the selection of individuals with OSA, their diagnosis was performed using polysomnography. For the diagnosis, the following criteria were considered: an apnea-hypopnea index (AHI) of ≥ 5, oxygen desaturation of ≥ 4%, and a reduction in continuous airflow for more than 10 seconds. The exclusion criteria included individuals suffering from infectious conditions, diabetes, smoking habits, neurological or psychiatric diseases, or morbid obesity (a BMI ≥ 40). Also excluded were those treated with continuous positive airway pressure (CPAP) or bilevel positive airway pressure (BPAP), those who had received pharmacological or surgical treatment for OSA, those with autoimmune disorders, acute respiratory conditions, and pregnant individuals. Furthermore, individuals undergoing treatment with antibiotics, corticosteroids, anticonvulsants, and antiepileptics within the past 6 months or before the clinical examination for the diagnosis of periodontal disease were also excluded.

### DNA Extraction and Genotyping

DNA extraction was performed from saliva samples obtained at the time of the periodontal examination, which were collected using the Oragene DNA Sample Collection Kit (OG-500 DNA Self-Collection Kit, Genotek, Ottawa, Ontario, Canada), according to the manufacturer's recommendations. Participants were required not to have consumed food or drink for at least 30 minutes before saliva collection. If this was not adhered to, they were asked to rinse their mouth with water and wait at least 15 minutes before donating the sample. For saliva collection, subjects were asked to gently rub their tongue against the inside of their mouth for 15 seconds and provide a quantity of saliva (without bubbles) up to the full mark on the collection vial. The vial was then closed, labeled, and gently inverted 10 times to mix the saliva with the storage and preservation solution of the kit. DNA was extracted from 0.5 mL of the sample using PT-L2P reagent (DNA Genotek, Inc., Ottawa, ON, Canada) according to the manufacturer's protocol and stored at -20°C. DNA quantity and quality were assessed by spectrophotometry using a NanoDrop 2000 (Thermo Scientific) and electrophoresis in 1.5% agarose gels. Segments of selected regions were amplified by endpoint polymerase chain reaction (PCR), followed by purification and genotyping of the SNVs of the genes of interest through Sanger sequencing. ( Table 1 ).

**Table 1:** Primers of selected genetic variants (SNV)

Gene	SNV	Forward primer	Reverse primer	PCR conditions
*IL-1β*	rs16944 A/G	CTCTGCCCCAGCCAAGAAAG	GCTGAAGAGAATCCCAGAGCA	D: 95°C for 3 minute, 34 cycles: 95°C 30s. AT: 59.2°C 40s E: 72°C x 1 minute, 5 minute
*IL-1β*	rs1143627 G > A,C,T	CTCTGCCCCAGCCAAGAAAG	GCTGAAGAGAATCCCAGAGCA	D: 95°C for 3 minute, 34 cycles: 95°C 30s. AT: 57°C 30s E: 72°C x 1 minute, 5 minute
IL-10	rs1800871 T/C	CCAGATATCTGAAGAAGTCCTG	TGGGGGAAGTGGGTAAGAGT	D: 95°C for 3 minute, 34 cycles: 95°C 30s. AT: 55.4°C 30s E: 72°C x 1 minute, 5 minute
IL-10	rs1800872 A/C	GGTGAGCACTACCTGACTAGC	CCTAGGTCACAGTGACGTGG	D: 95°C for 3 minute, 34 cycles: 95°C 30s. AT: 55.4°C 30s E: 72°C x 1 minute, 5 minute
IL-10	rs1800896 A/G	CCAGATATCTGAAGAAGTCCTG	CTCTTACCTATCCCTACTTCC	D: 95°C for 3 minute, 34 cycles: 95°C 30s. AT: 57.9°C 30s E: 72°C x 1 minute, 5 minute
IL17	rs2275913 G/A	AACAAGTAAGAATGAAAAGAGGACATGGT	CCCCCAATGAGGTCATAGAAGAATC	D: 95°C for 3 minute, 34 cycles: 95°C 30s. AT: 60.3°C 30s E: 72°C x 1 minute, 5 minute
IL-17	rs763780 T/C	TGAAGTGGAGGGAATTGGGG	GAATTCCGTTCCCATCCAGC	D: 95°C for 3 minute, 34 cycles: 95°C 30s. AT: 55.5°C 30s E: 72°C x 1 minute, 5 minute

PCR primers were designed using the Primer-BLAST tool from the National Center for Biotechnology Information (NCBI) website and synthesized at Macrogen Inc. (Seoul, South Korea). All genotyping assays were validated before processing the study samples. Electrophoresis on 2% agarose gels confirmed the quality of all PCR products. The SNVs of the purified amplicons were determined by Sanger sequencing (BigDye Terminator Cycle Sequencing Ready Reaction Kit; Thermo Fisher Scientific, Waltham, MA, USA; catalog no. 4337455).

Abbreviations: D: Denaturation; AT: Annealing Temperature; E: Extension.

### Statistical Analysis

The Kolmogorov-Smirnov test was used to select statistical tests to assess data normality. Sociodemographic variables and the distribution of diagnoses according to the severity of PD and OSA were analyzed for descriptive statistics. Allele and genotype frequencies were calculated in cases and controls, and for each subgroup of cases. In addition, Hardy-Weinberg (HW) equilibrium was calculated for each outcome group. A p-value < 0.05 was considered statistically significant. The association between SNVs and the risk of susceptibility to PD, OSA, and PD + OSA was determined by Chi-Square with Yates correction, Fisher's exact test, and Odds Ratio (OR) using 95% confidence intervals.

Data analysis was performed using IBM SPSS Statistics, version 25.0 (IBM Corp., Armonk, NY, USA). After performing univariate analyses and confirming the absence of collinearity, a multinomial logistic regression model was constructed. In this model, the dependent variable was disease type, categorized as periodontal disease (PD), obstructive sleep apnea (OSA), and PD/OSA comorbidity. Independent variables included sex, age, body mass index (BMI), and hypertension, with the control group serving as the reference category.

## Results

### Descriptive Data of the Study Sample

The sample consisted of 129 individuals from the city of Bogotá, with a control group of 64 individuals, and 65 individuals in the case group (18 with PD, 19 with OSA, and 28 with PD + OSA). The age range was between 30 and 70 years for both groups, with an average of ~ 46 years. The female sex was the most prevalent (54%). There is an imbalance in the proportion of men and women. Men constitute approximately one-third of the sample in the control and OSA groups, whereas they represent nearly two-thirds of the sample in the PD and PD/OSA groups. The majority of individuals in all groups were overweight and did not have hypertension. In the OSA group, 44% of participants presented with hypertension; however, compared with the control group, this difference was no statistically significant ( *p* = 0.0632), although the p value was close to the threshold for significance. Increasing the sample size might help clarify this association.

For all three case subgroups, the most prevalent diagnoses were severe chronic periodontitis (13%), mild OSA (13%), and a combination of severe chronic periodontitis with severe OSA (11%). See Table 2 .

**Table 2:** Demographic Features of the study subjects

Variables	Control	PD	OSA	PD + OSA
Age	45 ± 13	43 ± 13	53 ± 11	49 ± 12
Male Sex	41%	45%	55%	53%
Female Sex	59%	55%	45%	47%
BMI Normal weight*	45% Overweight 55%*	47% Overweight 53%*	55% Overweight 45%*	60% Overweight 10% Obesity 30%*
Arterial Hypertension	13%	21%	44%	13%

Multivariate logistic regression analysis revealed no statistically significant differences in most of the variables included in the model. Significant associations were only identified for two predictors: body mass index (BMI) and sex. Regarding BMI, although the control group showed a higher percentage (~ 46%) compared with the other groups (which ranged between 30% and 40%), the difference did not reach statistical significance when compared with the reference group. Regarding sex, an imbalance in the distribution between groups was observed, which suggests caution when interpreting its influence on the results. The overall model was statistically significant (χ ^2^ = 47.3; *p* = 0.001), indicating an overall better fit than the null model. The Pearson goodness-of-fit (χ ^2^ = 211.3, p > 0.05) and deviance (χ ^2^ = 170.2, p > 0.05) tests were not significant, supporting the model's suitability by not showing systematic discrepancies between observed and predicted values. Furthermore, the Cox and Snell pseudo-R ^2^ and Nagelkerke pseudo-R ^2^ , with values close to 0.45, indicate that the model explains ~ 45% of the variability in the dependent variable. The likelihood ratio analysis for the reduced model identified sex and BMI as statistically significant predictors of disease group membership. Specifically, the coefficient for male sex (b = 1.683; *p* = 0.012) was positive and significant, indicating that, compared with the control group, being male significantly increases the likelihood of belonging to the PD/OSAS comorbidity group.

### Genotyping Analysis

All groups were in Hardy-Weinberg equilibrium for all SNVs, including IL-1B (rs1143627), IL-10 (rs1800896), and IL-17 (rs2275913). After adjustment for sex, age, and BMI, multiple comparison tests showed statistically significant associations for both IL-1B SNVs (rs16944 and rs1143627) in individuals with OSA (rs16944: OR = 3.36, 95% CI: 0.3-2.3, p = 0.008; rs1143627: OR = 3.6, 95% CI: 1.5-8.5, p = 0.004) and PD + OSA (rs16944: OR = 4.8, 95% CI: 1.8-10.3, p = 0.00079; rs1143627: OR = 4.8, 95% CI: 2.2-10.4, p = 0.00007). The SNVs rs1800896 (IL-10) and rs2275913 (IL-17) also showed statistically significant associations with OSA. The other SNVs evaluated did not show a statistically significant association for any group ( Table 3 , Fig. 1 ).

**Table 3:** Comparison of SNVs in IL-1B, IL-10, and IL-17 Genes between the Control Group and Each Case Group (PD, OSA, and PD + OSA)

Gene	SNV	Control vs Group	Odds ratio	[95% CI]	*P* -Value*
IL-1 *B*	rs16944	PD OSA PD + OSA	0.94 3.36 4.8	(0.3–2.3) (0.3–2.3) (1.8–10.3)	0.918 0.008* 0.00079*
IL-1 *B*	rs1143627	PD OSA PD + OSA	1.3 3.6 4.8	(0.46–3.99) (1.5–8.5) (2.2–10.4)	0.758 0.004* 0.00007*
IL-10	rs1800872	PD OSA PD + OSA	1.6440 1.48 0.5492	0.850–3.182 0.747–2.930 0.264–1.144	0.069820 0.1303 0.054796
IL-10	rs1800896	PD OSA PD + OSA	0.6115 4.44 0.6449	0.263–1.4250 1.476–13.360 0.294–1.413	0.127185 0.004* 0.136526
IL-17	rs2275913	PD OSA PD + OSA	1.0610 0.2128 1.1199	0.564–1.995 0.087–0.520 0.6–2092	0.184030 0.000343* 0.361220
IL-17	rs763780	PD OSA PD + OSA	2.4632 0.7906 0.4852	0.462–13.140 0.244–2.562 0.117–2.021	0.145629 0.347684 0.160294

* Chi-square test with Yates' correction (post hoc multiple comparison test); significance level: p < 0.05.

**Fig. 1:**
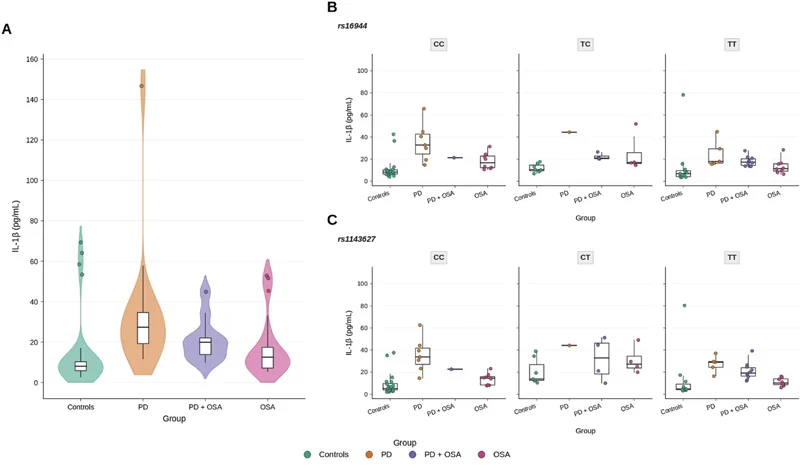
Comparison between groups for SNVs in the IL-1B gene. The SNVs rs1143627 and rs16944 showed statistically significant associations in individuals with OSA and with PD + OSA.

## Discussion

Periodontal disease (PD) and obstructive sleep apnea (OSA) share a systemic inflammatory background, which has led to the hypothesis of a possible bidirectional relationship between both conditions. However, despite epidemiological overlap, the molecular mechanisms underlying this association remain poorly understood, and the specific genetic factors contributing to the convergence of these diseases have not been fully established. In particular, there is a lack of studies addressing inflammatory genetic variants that may predispose individuals to the isolated or simultaneous occurrence of PD and OSA. Identifying such variants is essential to better understand the deregulation of inflammatory pathways involved in both conditions.

In this context, the present study investigated the association between selected single-nucleotide variants (SNVs) in *IL-1B* , *IL-10* , and *IL-17* genes and the presence of PD, OSA, or their coexistence. The results demonstrated statistically significant associations for both analyzed *IL-1B* SNVs (rs16944 and rs1143627) in individuals with OSA and in those with PD + OSA. Specifically, carriers of rs1143627 showed an increased risk of OSA, while rs16944 was associated with an increased risk of PD + OSA. These findings support a potential role for *IL-1B* –mediated inflammatory pathways in the shared pathophysiology of these conditions.

In the present study, most individuals with OSA and controls were overweight. Obesity, periodontitis, and OSA all exhibit increased activation of innate immune cells—particularly monocytes and macrophages—with a shift toward a pro-inflammatory phenotype. This is accompanied by elevated oxidative stress, which amplifies tissue damage in both the oral cavity and the upper airway. The resulting systemic inflammation predisposes individuals with OSA to cardiovascular and metabolic diseases. 37 38 39 Additionally, mediators such as TNF-α, IL-6, and IL-1β, which play central roles in the pathogenesis of both periodontal disease and OSA, also contribute to metabolic and cardiovascular complications in obesity. Adipose tissue dysfunction and immune cell infiltration in obesity lead to a systemic pro-inflammatory state, with elevated cytokines and chemokines that exacerbate inflammation and metabolic dysfunction. Obesity amplifies these effects, creating a synergistic cycle of inflammation and tissue injury.

Multivariate logistic regression revealed that, among the analyzed variables, only sex and body mass index (BMI) showed significant associations with disease group membership. The overall model demonstrated good fit and explanatory power, accounting for ~ 45% of the variance in the dependent variable. Specifically, male sex was identified as a significant predictor, increasing the likelihood of belonging to the PD/OSA comorbidity group. However, the relatively small sample size and the imbalance in sex distribution may have reduced statistical power and limited the generalizability of the findings. As an exploratory, hypothesis-generating study, the results should be interpreted with caution. Larger, well-balanced cohorts are required to validate these associations and to explore potential sex-specific effects.

Although obesity is known to exacerbate inflammation and metabolic dysfunction, intermittent hypoxia in OSA further intensifies oxidative stress and inflammatory signaling. Likewise, periodontitis contributes to systemic inflammation through the release of bacterial products and cytokines into circulation. However, the specific role of being overweight has not been established due to overlapping risk factors and confounding variables such as age, sex, diabetes, and lifestyle factors. In the present study, the increase in BMI did not appear to affect IL-1beta expression, suggesting that the association between IL-1beta and OSA may be more closely related to inflammation and hypoxia. This finding is consistent with previous studies demonstrating increased NLRP3 inflammasome activation and IL-1β release in patients with severe OSA, correlating with apnea–hypopnea and hypoxemia indices. 40 While the role of IL-1β in the pathophysiology of PD + OSA has been scarcely studied, its function in the pathogenesis of periodontal disease has been well demonstrated. Periodontal pathogens induce inflammasome activation and promote IL-1β maturation by activating caspase-1 (IL-1β converting enzyme, ICE). 41 Moreover, elevated IL-1β levels and increased plasma tissue factor have been observed alongside systemic inflammatory comorbidities, linking intermittent hypoxia to a systemic inflammatory response. Although periodontitis and OSA are both chronic diseases, the genes involved in the combined condition (PD + OSA) have not yet been elucidated.

After adjustment for sex, age, and BMI, in the group of individuals with both conditions, PD + OSA, only IL-1B showed a statistically significant association. The lack of adjustment for these factors is not only a methodological limitation, but also compromises the internal validity of the study, since the reported association could reflect common epidemiological factors rather than a true genetic relationship; without this adjustment, the results must be interpreted as crude associations that require confirmation in controlled analyses.

The SNV rs16944 in the IL-1B gene was statistically significant in individuals with OSA (OR = 3.36, 95% CI: 0.3-2.3; p = 0.008) and those with PD + OSA (OR = 4.8, 95% CI: 1.8-10.3; p = 0.00079). This SNV has previously been associated with PD in Chinese, Indian, Chilean, and Brazilian populations.42,44 Similarly, the SNV rs1143627 in the IL-1B gene showed statistically significant associations in the OSA group (OR = 3.6, 95% CI: 1.5-8.5; p = 0.004) and in the PD + OSA group (OR = 4.8, 95% CI: 2.2-10.4; p = 0.00007), and has been associated with PD in Asian and Caucasian populations. 42 In contrast, in individuals with OSA alone, statistically significant associations were observed for IL-10 and IL-17. Individuals with the rs1800896 (IL-10) polymorphism had a 4.44 times higher risk for OSA ( *p* = 0.004; OR:4.44 CI: 1.476–13.360). This SNV has also been associated with PD in both Caucasian and Asian individuals. 43 44 IL-10 is an anti-inflammatory cytokine with immunomodulatory properties. In individuals with OSA, the role of IL-10 has been reported both as a risk factor and as a protective agent against excessive inflammation, suggesting that IL-10 could potentially serve as a diagnostic biomarker for OSA. Nevertheless, the findings related to the role of IL-10 in OSA are inconsistent. The role of the anti-inflammatory IL-10 in OSA diagnosis requires further investigation, perhaps in larger populations. 45 46

In the present study, the SNV rs2275913 in the IL-17 gene showed a statistically significant difference in the OSA group. However, this was associated with a protective effect for OSA (OR = 0.218, 95% CI: 0.087-0.520; p = 0.00343). Interestingly, the IL-17 rs2275913 polymorphism has been associated with periodontitis susceptibility, conferring a protective effect in Asian populations. This association is particularly evident in Asian populations IL-17 has protective roles in mucosal immunity, its dysregulation leads to chronic inflammation and bone loss, highlighting its dual role in oral and systemic diseases. However, the precise causal relationships and therapeutic implications in OSA and periodontitis remain areas of active investigation. 47 Several investigations support a relationship between elevated IL-17 and the presence or severity of OSA, but one meta-analysis reported no significant difference in IL-17 levels 42 in these individuals. The cytokine IL-17 is predominantly produced by Th17 cells, a type of immune cell that contributes to inflammatory and autoimmune responses. In adults with OSA, IL-17 has been associated with promoting inflammation in the airways, which can exacerbate the symptoms and severity of OSA. Increased IL-17 levels are thought to contribute to the pathogenesis of OSA by enhancing inflammatory responses, which could lead to airway remodelling and increased airway resistance, both key features of OSA. 43 Moreover, genetic polymorphisms in the IL-17 gene have been studied for their potential to influence IL-17 production and activity, thereby impacting the development and severity of OSA. These genetic variations can affect how much IL-17 is produced in response to inflammatory stimuli, which in turn can influence the inflammatory state associated with OSA. 30

Periodontitis and OSA share overlapping risk factors and inflammatory mediators, with IL-17 acting as a central link; however, its specific role in individuals with PD + OSA has not been sufficiently studied. 48 in individuals with PD + OSA has not been sufficiently studied. Polymorphisms in the IL-17 gene promote the recruitment of neutrophils and the production of other pro-inflammatory cytokines, exacerbating the inflammatory response. This process can lead to the breakdown of the connective tissue and bone that support teeth. Nonetheless, IL-17 plays an essential role in defending against the bacteria that cause periodontitis, highlighting its dual role in both promoting disease progression and participating in the immune response to infection. Furthermore, IL-17 has been linked to the regulation of bone metabolism, influencing the activity of osteoclasts, the cells responsible for bone resorption, through mechanisms involving RANKL (receptor activator of nuclear factor kappa-Β ligand), a key regulator of bone remodeling. Depending on the balance of immune responses in the local environment, IL-17 activity can either protect against or contribute to bone loss. Overall, the action of IL-17 in periodontitis, and possibly in OSA, appears to be influenced significantly by disease context and the stage, suggesting that its effects are not linear but context-dependent, with both protective and destructive outcomes determined by the periodontal microenvironment. 48 49 50 However, the dual role of IL-17—protective in mucosal immunity but pathogenic when dysregulated—underscores the complexity of targeting this cytokine therapeutically. 51 The interplay between IL-17, the oral microbiome, and systemic inflammation suggest that interventions modulating IL-17 or its upstream regulators (e.g., IL-23, D-resolvins, IL-35) could have therapeutic value, but more clinical research is needed. 52

In conclusion, the present study suggests a potential association between selected polymorphisms in the IL-1beta, IL-10, and IL-17 genes and the coexistence of PD and OSA, supporting the hypothesis that cytokine-mediated inflammatory pathways may be involved in PD-OSA comorbidity. However, these findings should be interpreted with caution.

Several limitations must be acknowledged. The relatively small sample size and the imbalance in subgroup distribution reduced statistical power and limited the ability to explore associations with other systemic inflammatory comorbidities, particularly cardiovascular and metabolic disorders that frequently coexist with PD and OSA. In addition, the restricted sample precluded stratified analyses according to age, sex, and body mass index—factors known to influence inflammatory responses. The cross-sectional design further limits causal inference, allowing the identification of associations but not temporal or directional relationships. Moreover, only a limited number of single-nucleotide variants were evaluated for each interleukin gene, and unexamined variants, gene–gene interactions, or epigenetic mechanisms may also contribute to disease susceptibility.

Taken together, these results should be viewed as exploratory and hypothesis-generating, providing preliminary insight into the inflammatory genetic background of PD–OSA comorbidity. Larger, well-powered, longitudinal, and multi-omic studies are warranted to validate these associations, clarify gene–environment interactions, and advance the identification of reliable biomarkers and potential therapeutic targets for these overlapping chronic conditions.
